# Reporting the use of AI in horizon scanning: a brief communication on methodological standards

**DOI:** 10.1017/S026646232610405X

**Published:** 2026-07-29

**Authors:** Hannah O’Keefe, Claire Eastaugh, Chris Marshall

**Affiliations:** 1 Population Health Sciences Institute, Newcastle University, Newcastle upon Tyne UK; 2 NIHR Innovation Observatory, Newcastle University, Newcastle upon Tyne, UK

**Keywords:** horizon scanning, reporting items, artificial intelligence, health and social care, life sciences

## Abstract

**Objectives:**

The UK is being positioned to become a leader in the field of artificial intelligence (AI), with the government driving the AI Opportunities Action Plan. In line with the government’s push for an AI-driven future, horizon-scanning organizations are leveraging AI and embedding it within their methodological practices. To ensure the transparent, reliable, and responsible use of AI, horizon scanning requires a set of tailored reporting items. However, this is a notable gap in the current methodological landscape.

**Methods:**

This methods communication discusses the current guidelines, policies, and statements around the use of AI relevant to futures and foresight research for health, social care, and life sciences policy and practice. We include stakeholder positions, research funding applications and writing for publication within the discussion and demonstrate how these guidelines relate to horizon scanning practices, with a focus on the common recommendations for reporting AI.

**Results:**

We have utilized the documents to identify and consolidate reporting recommendations into a minimal set of reporting items for AI in horizon scanning. A total of 139 data points were consolidated into 46 reporting items under six categories.

**Conclusions:**

Further work is needed to validate these reporting items, but this interim step toward a robust reporting framework fills an immediate methodological need. We encourage those working in the horizon scanning space to continue to explore and embed AI transparently and responsibly in this rapidly adapting field.

## Background

The UK is positioning itself as a world leader in artificial intelligence (AI), currently ranking 4th in the Global AI Index tables (1). The UK scores highly for the availability of skilled practitioners, regulatory context of AI use, and government commitment to AI, with research and innovation at the forefront of UK outputs (1;2). In January 2025, the British government released the AI Opportunities Action Plan (3). The plan aims to grow Britain’s already strong foundations through three core goals: to invest in sufficient, secure, and sustainable AI infrastructure; train and upskill the workforce to lead in AI development; and reduce the cross-economy divide between public and private sector adoption of AI (3). There is a 50-point breakdown of steps required to fulfil these goals and an expectation that, should they be met, the UK could see economic growth of £400 billion by 2030 (4).

As AI becomes increasingly embedded across research, healthcare, and everyday systems, guidelines and policies are evolving to ensure its rigorous and responsible use. This is particularly important in evidence generation that will inform policy and practice for our health and social care services and life sciences industries. Horizon scanning is no exception to this, as methodologies aim to capture insights and early trends over the emerging, transitional, and imminent horizons, which support market access pipelines for medical technologies and therapeutics (5–7). Horizon scanning is a subset of futures and foresight methodologies, and work in this field is underway to automate high-burden tasks such as data ingestion, integration across multiple sources, and screening, providing scalable foundations for generating timely insights and intelligence to inform stakeholder decision making. Thus, futures and foresight organizations should engage with published government and stakeholder guidance where applicable, across all aspects of the research lifecycle. The use of AI should be reported in a clear and transparent way, consistent with available guidance, to minimize ambiguity.

Previous work has shown that reporting in horizon scanning is highly variable and would benefit from greater standardization (5). This need is becoming more acute with the introduction of AI, and many existing reporting standards are now being revised with AI-specific extensions across different fields (8;9). However, only two have been formally released in the related fields of systematic reviewing and health economics, and those in development are not directly transferable to horizon scanning or broader futures and foresight analysis (9;10). This is largely due to reporting standards being tailored to follow the strict methodological steps involved in systematic reviewing. In horizon scanning, these steps are similar but perhaps less rigid. As horizon scanning adapts according to whether you are seeking disruptive or long-term horizons, the definitive methodological steps are dependent on the type of scan being conducted. Nevertheless, systematic reviewing is arguably the most closely related discipline to horizon scanning; thus, we have drawn heavily on documentation from this field to inform this work. We therefore reviewed reporting recommendations in current guidelines, policies, and statements relevant to futures and foresight analyses, including stakeholder positions, funding applications and publication requirements, and consolidated them into a minimal set of reporting items for horizon scanning projects.

## Methods

### Information sources

Known guidelines and policy documents from core NIHR Innovation Observatory horizon scanning center stakeholders were reviewed for evidence of reporting recommendations with relevance to AI use in horizon scanning. An information retrieval specialist identified secondary documents from innovators in the field of evidence synthesis automation, funders and publishers through grey literature searches and bidirectional citation chaining. Grey literature searches were conducted in Google and Google Scholar using a pragmatic approach. Terms used for searching were: AI guidelines; reporting AI use; acknowledging AI use; AI use in publications. No limitations were placed on these searches. Given the academic search history of the information specialist, it was not deemed necessary to use specialist measures to avoid search engine biases for this literature review as would usually be done in a systematic review. Explicit inclusion and exclusion criteria were not applied to this literature review. However, the authors purposefully selected documentation that had an academic, policy, or publication remit and was reflective of the needs of evidence synthesis landscape. Again, these documents were reviewed for recommendations relating to the reporting of AI use.

### Data extraction and synthesis

Blocks of text related to reporting were extracted verbatim into Excel, and the section of the document containing the information was noted. These blocks of text were narratively synthesized in the first instance to identify categories. The extracted text was then scrutinized, and similar or repetitive data points were consolidated to formulate a list of uniform reporting items. These items were then grouped into the broad categories identified previously. Data synthesis, reduction/consolidation, and categorization were checked by a second author for accuracy.

## Results

We identified 14 core documents to review across the four areas; each document is discussed individually below. We used the information presented in each of the 14 documents plus the Algorithmic Transparency Recording Standard (ATRS) v3.0 to isolate a list of potential reporting items. The ATRS is a UK government reporting standard for public sector organizations, which enable the transparent reporting of algorithmic tools.

We isolated 139 data points in total (Supplement 1) and consolidated them to produce a minimal set of reporting items.

### Guidance documents

We extracted 111 of the 139 data points from 6 documents: Responsible AI in Evidence SynthEsis (RAISE): guidance and recommendations; PRISMA-trAIce; Guidance to civil servants on the use of AI; the Department for Science, Innovation and Technology (DSIT) guide to using AI in the public sector; Government Digital Services – *Artificial Intelligence Playbook for the UK Government*; ATRS v3.0.

RAISE outlines a total of 87 recommendations across the five roles (11). The most extensive recommendations fall under the AI model development teams (n = 26), 11 of which are specific to reporting. A further four reporting-based items were identified from the remaining roles (11).

ATRS v3.0 was by far the most extensive, with 65 data points extracted, accounting for 46 percent of the total data. Seventeen items were extracted from PRISMA-trAIce, one each from the Civil Servants’ Guide and DSIT, and 12 from the *Artificial Intelligence Playbook for the UK Government*.

### Position statements

We used a single position statement from the National Institute of Health and Care Excellence (NICE). Only five data points were retrieved in relation to reporting AI use, accounting for ~3 percent of the total data.

### Funding application guidance

We purposefully selected the Research Funders Policy Group’s Joint Funders Statement and the three member organizations with individual AI statements: Wellcome, Cancer Research UK (CRUK), and UK Research and Innovation (UKRI). We extracted two data points from each of the organizational statements.

### Publisher guidelines

We have selected four publishers known for accepting horizon-scanning research papers in one or more of their journals and reviewed their current guidelines: Frontiers; Wiley; BMJ; and Cambridge University Press.

Data extraction from Frontiers and Wiley produced five data points each. Four were from BMJ, and only one from Cambridge University Press.

### Minimal set of reporting items

Data points were consolidated into 46 interim reporting items across six categories: system/tool/platform details (*n* = 14); justifications (*n* = 9); risk and mitigation (*n* = 3); data processing (*n* = 11); process and validation (*n* = 7); report writing (*n* = 2). We present this as a minimal set of reporting items in Table 1.

**Table 1.** Minimal set of reporting items for the use of artificial intelligence in horizon scanning Table 1. long description.

Topic	Reporting item	Page	Comments
System/tool/platform details	Name of system/tool/platform		
	System/tool/platform ID (if applicable)		
	Version number of system/tool/platform		
	URL for system/tool/platform		
	URL for guidance documentation		
	Development phase of system/tool/platform		
	System/tool/platform development history		
	Citations of training/testing/validation		
	Owning organization type (e.g., university, company)		
	Organization contact information (e.g., email address, preferably team rather than individual)		
	Sources of funding		
	Intended purpose of system/tool/platform		
	Function and capability of system/tool/platform		
	List models within the system/tool/platform		
Justifications	Justification and purpose of using AI (including scale and frequency of use)		
	Which parts of the project has AI been used for (signpost in the main text)		
	Was AI use pre-defined in a protocol (signpost to protocol)		
	What are the benefits of using the system/tool/platform (including details of previous processes)		
	Who does the outcome affect (e.g., specific region, population)		
	Who was involved in using the system/tool/platform (including organisation, senior responsible person, job title, and contact details)		
	What are the appeal and review processes for decision outcomes, where applicable		
	Was training required (including details of training provided)		
	Where external suppliers involved (including name, company, contact details, Companies House number, and short description of role)		
	What was the external supplier procurement procedure (if required)		
Risk and mitigation	Data risks (including equity and biases)		
	System risks (including equity and biases)		
	Risk mitigation strategy		
Data processing	Data collection (including dates, sources, and search strategies)		
	Data set description (including summary, type of data, size of data set)		
	Data labelling and annotation		
	Data cleaning		
	Missing data		
	Sensitive data attributes (e.g., personal data, protected characteristics, proxy variables)		
	If data has been included/excluded using the AI, has this been recorded (signpost to main text)		
	Justification for using sensitive data (including suitability assessment name and link to output of assessment)		
	Has confidential information been removed (including details of redaction or cleaning process)		
	Data storage and sharing (including date archived and updated)		
	Data access requirements		
	Impact assessments (e.g., data protection impact assessments (DPAIS), equality impact assessments, algorithmic impact assessments)		
Process and validation	Date of use		
	Date updated (including any known planned updates)		
	Prompt engineering steps (including development, validation, and limitations)		
	Strengths and limitations of the system/tool/platform		
	Ethical or lawful considerations (including privacy and copyright)		
	What assessment or validation was used		
	Validation inputs/outputs (including performance metrics – precision, recall, F1; privacy metrics, computational efficiency metrics, performance thresholds)		
	Have the AI outputs been reviewed by humans (including details of the process)		
Reporting	Has generative AI been used to improve the language of this report (including details of how)		
	Does the main text contain a disclosure or acknowledgment of AI use		

## Discussion

### Guidance documents

To date, no guidance documents have been released explicitly for horizon scanning or broader futures and foresight analyses. However, collaborative efforts have been made for AI in the evidence synthesis community, a closely aligned methodology. Additionally, broader guidance has been written by DHSC around the use of AI in the public sector.

An established community of evidence synthesis and systematic review experts is developing the RAISE guidance and recommendations (11). The authors highlight the responsibilities of the five main roles across the systematic reviews and evidence synthesis ecosystem. These roles are discussed individually, yet authors acknowledge that people and organizations may have multiple roles in the evidence synthesis lifecycle (11).

The evidence synthesis community has a responsibility to ensure that best practice guidelines and standards are followed and that evidence syntheses are produced to a high standard. Authors of RAISE report on the need to publicly and clearly report on the development of the AI models, including versions, purpose and planned future updates. Second, to report on the underlying system and avoid unnecessary use of commercial confidentiality. This includes what data was used to train the model, how the model was evaluated, and what steps were taken to validate the model (11). There is a push toward the open-source movement, with recommendations to make training data easily accessible. However, challenges arise with proprietary models where companies often cloud access to training data and back-end processes (such as internal word-by-word weighting or hidden reasoning traces) as commercially sensitive and confidential. Intellectual property rights and security are commonly cited as a justification for this. As with all research, funding sources, conflicts of interest, strengths, limitations, equity issues, and biases should all be reported. Authors suggest detailing potential biases in training data and outlining the scenarios that the model is best suited for to enable users to make informed decisions about if and when to apply the model to their own work. Finally, there is a strong recommendation to provide a comprehensive user guide. Such guides will allow evidence synthesis methodologists, one of the five core roles denoted in RAISE, to effectively evaluate the applicability and reliability of the AI model. Elsewhere in the document, reporting is mentioned in relation to following PRISMA guidelines (a long-standing and well-documented set of reporting standards for evidence synthesis) and reporting evaluations of AI tools (11). Currently, PRISMA-trAIce is the only known published reporting standard for AI use in systematic reviews (9). It should be highlighted that this standard has not been registered with or endorsed by PRISMA (12), nor has it been registered with the EQUATOR network. Thus, despite using the PRISMA name, it is not an official extension. Nevertheless, the reporting items within this publication are broadly reflective of those in the RAISE guidelines. These reporting items are outlined by heading within the review, that is, title, abstract, introduction, methods, results, and discussion. Although this is a valid and preferred structure for systematic reviews, it may not hold true for horizon scanning reports, which are often structured differently.

DHSC, under the Conservative Party government, released several guides surrounding AI. Two of relevance to futures and foresight research are the guidance to civil servants on the use of AI and the DSIT guide to using AI in the public sector (13;14). Although these provide a grounding for considering the appropriateness of using AI for a given task, neither provides a comprehensive overview of reporting. The only mention of reporting is in the civil servants’ guide, which states it is important to make readers aware that an AI model has been used and that this should be done by citing the model and its URL in a footnote, alongside any sources that were input to the model. This approach is contradictory to the recommendations outlined in RAISE, which aligns with a more formal and comprehensive approach to reporting on the use of AI.

Most recently, under the labor government, the guidance has been superseded by the Government Digital Services – *Artificial Intelligence Playbook for the UK Government* (15). This holds a far more extensive description of reporting for transparency and explainability. One of the primary recommendations from this section is the use of ATRS (16). This is a comprehensive form which considers metadata, an overview of the model for the public, and detailed information for interested parties comprising five sections. The reporting standard is available as a freely downloadable Excel template. The Playbook also refers to several additional standards and external resources which may be beneficial for reporting (15;17).

### Position statements

As with the above guidance documents, the NICE position statement on AI in evidence generation does not directly address horizon scanning and futures and foresight analyses, but it does hold relevance through its section on systematic reviewing and evidence synthesis (18). The statement aims to outline three core areas for AI in research: the expectations that NICE has when AI is used; existing regulations, good practice, standards, and guidelines that should be followed; and support for committee members and external assessment groups in understanding and critiquing AI methods. Sixteen broad coverage points are given to outline the NICE position, with further points broken down by types of evidence generation (18).

In the generalized points, reporting criteria are mentioned five times. First, to highlight the need for clear documentation to be written following AI ethical frameworks (18). NICE suggests multiple frameworks that could act as a basis for reporting (14;19–21). The ethical application of AI is a wide-ranging topic. Many guides are primarily concerned with ensuring individuals provide informed consent for any use of personal data. Although general data protection regulation does not limit the use of confidential or protected data in AI systems, it does ensure the use is fully regulated and reported as such. Similarly, there is a cybersecurity risk associated with AI through data poisoning and prompt injection (22;23). Risks and mitigation strategies should be reported with evidence provided where appropriate. This is also applicable to methodological risks such as bias (24). Cochrane, PALISADE, TRIPOD+AI, and the ATRS are mentioned as established guidelines and checklists (16;25;26). NICE recommends adhering to these as a mechanism for reporting risk mitigations and providing transparency. NICE further states that organizations must declare the use of AI and give a rationale, regardless of data sources. Justifiable use cases should be reported in an accessible format with appropriate referencing, preferably using lay language. The remainder of the NICE documentation discusses possible use cases of AI and does not mention reporting any further (18).

### Funding application guidance

Funding applications are an integral part of the research process. UK funders require detailed outlines of the proposed work and justification for the monetary value requested. As with many institutions, funders are cautious about the use of AI in preparation and assessment of funding applications. The Research Funders Policy Group was established in 2019 to provide a cohesive approach to science and health research funding in the UK. It currently consists of eight member organizations: Association of Medical Research Charities (AMRC); British Heart Foundation (BHF); CRUK; National Institute for Health and Care Research (NIHR); Royal Academy of Engineering; Royal Society; UKRI; Wellcome. The Research Funders Policy Group has released a joint funders statement regarding AI, signed by all participant organizations (27). This statement outlines the minimum expectations when using AI to support the funding application process. It is a brief statement, highlighting the need for applicants to acknowledge if AI has been used in their application as best practice. It also states that peer reviewers should not use AI to aid their assessment due to confidentiality within the applications (27).

Of the eight member organizations, only three have released individual policies as of March 2026. The UKRI policy stipulates that sensitive or personal data should not be used in AI models without formal consent, as seen with the NICE guidance (28). Again, risk mitigation should be considered, particularly pertaining to any data that is confidential and used without informed consent, falsified, fabricated, plagiarized, or misrepresented. Although no direct reporting standard is given, the policy again highlights the need for transparent reporting of any use of AI within the application. There is an additional clause, above that outlined in the joint funders statement, asserting that peer reviewers must not take into account or speculate that AI has been used to develop the application within their assessment (28). Wellcome and CRUK have both published individual policies which echo the URKI outline (29;30).

### Publisher guidelines

Publishers are concerned about the spread of fabricated or plagiarized material that may come from using AI models to write or edit publications. Understandably, this has sparked a wave of publisher statements or guidelines about the use of AI. In all cases across Frontiers, Wiley, BMJ, and Cambridge University Press, the publisher states that AI does not meet the requirements for authorship and instead should be clearly and transparently reported in the methods, a disclosure, or acknowledgements as appropriate (31–34). The authors remain responsible for the content of the work and must take due diligence to ensure the work is accurate and avoids plagiarism. This is not only applicable to written work, but also to data, images, and visual representations. Frontiers clearly requests that prompts be listed in Supplementary Material to aid transparency (32). Similarly, BMJ asks authors to consider a summary of input, output, and how the output was reviewed to be added as supplementary information (31). Regarding peer-review, BMJ, Wiley, and Frontiers have explicitly stated that unpublished manuscripts should not be uploaded to AI models (31–33). This is due to confidentiality, as seen with funding applications. BMJ and Wiley do however allow peer reviewers and editors to use AI to improve the quality of their comments, provided it is reported when submitting the response (31–33).

## Limitations

This communication was developed by information retrieval and AI experts within the NIHR Innovation Observatory. A key limitation is that this is not a systematic review and does not consider guidelines or policies beyond health and social care. The proposed reporting items reflect the authors’ judgement and opinion rather than wider consensus. Although not exhaustive or validated through formal consensus methods, they provide a minimal reporting framework for futures and foresight projects, addressing a clear gap in horizon scanning practice (Table 1).

## Conclusions

To facilitate the reporting of AI in horizon scanning, we have isolated the statements referring to reporting from guidelines, statements, and policies applicable to futures and foresight research. We present a minimal set of reporting items for those working in the horizon scanning, futures, and foresight space. As the UK prepares to transform into a world-leading hub of AI, it is our responsibility as researchers to support this movement through embedding reporting processes as routine practice and ensuring the responsible and transparent use of AI.

## Supporting information

10.1017/S026646232610405X.sm001 O’keefe et al. supplementary material O’keefe et al. supplementary material

## Supplementary material

The supplementary material for this article can be found at http://doi.org/10.1017/S026646232610405X.

## Table 1. Long description

Minimal set of reporting items for the use of artificial intelligence in horizon scanning. The reporting items were generated from 139 data points that had been extracted from 15 documents. Authors intend for these to be used as interim reporting items until such time as a formal reporting standard is produced.

Navigate back to Table 1..
