# Supplementary material for: Reporting the use of AI in horizon scanning: a brief communication on methodological standards
Source: Int J Technol Assess Health Care. 2026 Jul 29;42(1):e68. doi: 10.1017/S026646232610405X (PMC13468941; doi:10.1017/S026646232610405X)
Supplement: Supplementary file 1 [file S026646232610405Xsup001.docx]

# Supplement 1: Data extraction table

Recommendations related to reporting of AI use extracted from documentation relevant to evidence synthesis.

| Reporting item | Source |
| --- | --- |
| In the report, the justification for the use of an AI system should be stated explicitly. This should include the steps taken to ensure it is appropriate for use in the context of that evidence synthesis and how the authors have determined that its use does not undermine the trustworthiness or reliability of its conclusions. | RAISE section1.2 |
| Evidence synthesis reporting should adhere to the Preferred Reporting Items for Systematic Reviews and Meta-Analyses (PRISMA),which include items on reporting automation tools used at different stages of the synthesis process. In general, evidence synthesists should report:   - the name(s)of the AI system(s), tool(s) or platform(s) version(s)and date(s)it was/ they were used - state which parts of the synthesis used AI, and how it was used - full details of how data in the review interacted with the AI tool, including where data were generated (whether human or AI) - cite or reference training and testing evaluations that justifies the use of the AI system in your synthesis - Declare funding sources and any financial and non-financial interests | RAISE Section 1.8 |
| If you validated an AI system for use in your evidence synthesis, make the make the input and outputs publicly available(for example, by including them as a supplementary file) | RAISE Section 1.9 |
| If you validated an AI system for use in your evidence synthesis, report any equity issues or biases within the AI systems, including what biases there may have been in the systems’ training data and strategies for users to help mitigate potential issues due to biases | RAISE Section 2.2 |
| Ensure full and transparent reporting of evaluations, including full methods and publishing null results. Full reporting should also include:   - the name of the AI system, version and date if was used - how and where the data were collected - any exclusion criteria that were applied(as this may result in bias in the evaluation) - how data were labelled or annotated as part of building the model - any processes used to clean the data, including handling missing data - prompt engineering and the documentation of how prompts are developed and validated(for AI using LLMsthat require prompts) | RAISE Section 2.7 & 3.6 |
| Publicly and clearly report details about the system, including name, version and purpose. Include information about how the model has developed over time and what future updates are planned. | RAISE Section 3.8 |
| Report how the system works; do not hide reasonable openness behind ‘commercial confidentiality’ where this is unnecessary | RAISE Section 3.9 |
| Be clear about which data are used to train, evaluate, and validate the tool, and where such data are stored and shared. | RAISE Section 3.10 |
| Provide clear guidance to users and assist them to be accountable for the use of your AI system. Comprehensive user guidance enhances transparency, facilitates accountability, promotes appropriate and ethical use, and ultimately helps evidence synthesists make informed decision about their use. | RAISE Section 3.11 |
| Report the strengths and limitations of the AI system(s), including what scenarios it is best suited to and when it may be inappropriate to use, with enough detail that users can decide whether it will be appropriate to use in their own work. | RAISE Section 3.12 |
| Report any equity issues or biases within the AI systems, including what biases there may have been in the systems training data and strategies for users to help mitigate potential issues due to biases | RAISE Section 3.13 |
| Be transparent about your funding sources and declare any financial and non-financial interests. | RAISE Section 3.14 |
| Consider reporting on the system’s development history, documenting not only data sources but errors discovered and what modifications were made | RAISE Section 3.16 |
| Clearly detail any ethical or lawful considerations that users of your AI system need to be aware of, for example, any that relate to areas such as privacy, copyright, etc | RAISE Section 3.25 |
| Report recommendations of AI systems which are suitable for particular tasks in your organisation. Where possible, list AI systems with endorsement for use in your evidence syntheses. | RAISE Section 4.2 |
| Whether using the outputs from generative AI either verbatim or with minor alterations, it is important to make clear to those reading that one of these tools has been used. To do this the tools should be cited in a footnote, with its URL and any sources used as inputs. | Guidance to civil servants on use of generative AI  Section Practicalities of using generative AI in your role:  References |
| It can be useful to keep a central record of all AI technologies you use, listing:   - where AI is in use - what the AI is used for - who’s involved - how it’s assessed or checked - what other teams rely on the technology | A guide to using artificial intelligence in the public sector  Section  Assessing if artificial intelligence is the right solution -  Recording accountability |
| ID | ATRS 3.0  Section Metadata |
| Standard version | ATRS 3.0  Section Metadata |
| Organisation type | ATRS 3.0  Section Metadata |
| Function | ATRS 3.0  Section Metadata |
| Capability | ATRS 3.0  Section Metadata |
| Task | ATRS 3.0  Section Metadata |
| Tags | ATRS 3.0  Section Metadata |
| Date of completion | ATRS 3.0  Section Metadata |
| Date updated | ATRS 3.0  Section Metadata |
| Date archived | ATRS 3.0  Section Metadata |
| Title: Provide the name used to identify the algorithmic tool. This will be used in the title for the algorithmic transparency record. | ATRS 3.0  Section Metadata |
| Organisation name: Provide the full name of the organisation, department or public sector body that completed the algorithmic transparency report and carries responsibility for use of the algorithmic tool. This name will be used in the title of the algorithmic transparency report | ATRS 3.0  Section Metadata |
| Phase: Indicate the phase the tool is currently in. | ATRS 3.0  Section Metadata |
| Region: Indicate the primary region over which the tool affects the public. | ATRS 3.0  Section Metadata |
| One sentence description: Provide a one sentence description of the tool. This will function as a subheading underneath the title of the record. | ATRS 3.0  Section Metadata |
| Name: Provide the name used to identify the algorithmic tool. | ATRS 3.0  Section Tier 1 |
| Description: Provide a basic overview of the purpose of the algorithmic tool. It should include:   - how the algorithmic tool is used - why the algorithmic tool is being used | ATRS 3.0  Section Tier 1 |
| Website URL: Provide a URL reference to a page with further information about the algorithmic tool and its use. This could be a local government page, a link to a GitHub repository or a departmental landing page with additional information. | ATRS 3.0  Section Tier 1 |
| Contact email: Provide an email address for the organisation or team responsible for this record. For continuity and security purposes, we advise using or creating a team email address instead of using an individual email address. | ATRS 3.0  Section Tier 1 |
| Organisation or department: Provide the full name of the organisation, department or public sector body that carries responsibility for use of the algorithmic tool. | ATRS 3.0  Section Tier 2.1 |
| Team: Provide the full name of the team that carries responsibility for use of the algorithmic tool. | ATRS 3.0  Section Tier 2.1 |
| Senior responsible owner: Provide the role title of the senior responsible owner for the algorithmic tool. | ATRS 3.0  Section Tier 2.1 |
| External supplier involvement: Have external suppliers been involved in the development and operation of the tool? | ATRS 3.0  Section Tier 2.1 |
| External supplier: Provide the name of any external organisation that has been contracted to develop the whole or parts of or the algorithmic tool. | ATRS 3.0  Section Tier 2.1 |
| Companies House Number: If available, provide the Companies House number of the external organisation that has been contracted to develop either the whole, or a part of the algorithmic tool. | ATRS 3.0  Section Tier 2.1 |
| External supplier role: Provide a short description of the role the external supplier had in the development of the algorithmic tool. If multiple organisations have been contracted or there are multiple companies involved in the delivery of the tool, these relationships should be described clearly and concisely. | ATRS 3.0  Section Tier 2.1 |
| Procurement procedure type: Provide the procurement procedure through which the contract has been set up.  For example, ‘open’, ‘restricted’, or ‘competitive procedure with negotiation’, or ‘call-off from a dynamic purchasing system’, and ‘call-off from a framework’ where a contracting authority has awarded to a supplier under a pre-established framework agreement. | ATRS 3.0  Section Tier 2.1 |
| Data access terms: Detail the terms of access to (government) data granted to the external supplier. | ATRS 3.0  Section Tier 2.1 |
| Detailed description: Provide a detailed description of how the algorithmic tool works. Compared to the high-level description in Tier 1, the detailed description should provide an explanation at a more granular and technical level. | ATRS 3.0  Section Tier 2.2 |
| Scope: Describe the purpose of the tool and the scenario(s) that it has been designed to be used in. This can include a list of potential scenarios that the tool was not designed for, which can help to avoid misconceptions about the scope and purpose of the tool. | ATRS 3.0  Section Tier 2.2 |
| Benefit: Describe the key benefits that the algorithmic tool is expected to deliver, and an expanded justification of why the tool is being used. | ATRS 3.0  Section Tier 2.2 |
| Previous process: Provide a description of the decision making process that took place prior to the deployment of the tool, where applicable. | ATRS 3.0  Section Tier 2.2 |
| Alternatives considered: Provide, where applicable, a list of other algorithmic and non-algorithmic alternatives considered. This can include why a certain algorithmic approach was chosen over other approaches, and why the chosen tool is the best available option given any known risks and shortcomings and trade-offs. | ATRS 3.0  Section Tier 2.2 |
| Process integration: Explain how the algorithmic tool is integrated into the decision-making process and what influence the algorithmic tool has on the decision-making process. Provide a more detailed description of the wider decision- making process into which the algorithmic tool is embedded. | ATRS 3.0  Section Tier 2.3 |
| Provided information: Describe how much and what information the algorithmic tool provides to the decision maker. For example, this can include the tool output and the form in which it is presented to the decision maker. | ATRS 3.0  Section Tier 2.3 |
| Frequency and scale of usage: Provide information on how regularly the algorithmic tool is being used and the scale of use.  For example, the number of decisions made per month, the number of citizens interacting with the tool, etc. | ATRS 3.0  Section Tier 2.3 |
| Human decisions and review: Describe the decisions that people take in the overall process. It should also detail human review options. For example, specific details on when and how a person reviews or checks the tool decision or prediction. | ATRS 3.0  Section Tier 2.3 |
| Required training: Describe the required training those deploying or using the algorithmic tool must undertake, if applicable. This can include training for operations, users, maintenance, compliance, or oversight personnel. | ATRS 3.0  Section Tier 2.3 |
| Appeals and review: Describe the mechanisms that are in place for review or appeal of the decision available to the general public, where applicable. | ATRS 3.0  Section Tier 2.3 |
| System architecture: Provide at least one of the following:   - A high-level written description of the system architecture, summarising the key technical features of the tool. - A URL reference to documentation about the tool architecture. For example, a link to a GitHub repository image or additional documentation about the system architecture | ATRS 3.0  Section Tier 2.4.1 |
| Phase: Indicate the phase the tool is currently in. | ATRS 3.0  Section Tier 2.4.1 |
| Maintenance: Provide details of the maintenance schedule and frequency of any reviews. This includes information such as how often and in what way the tool is being reviewed post-deployment, and how it is being maintained if further development is needed.  This can concern maintenance both by the supplier as well as by the operator/user of the tool. | ATRS 3.0  Section Tier 2.4.1 |
| Models: Provide a list of models that feature within the tool. These may be rule-based, machine learning, and other statistical or mathematical models. For example, a video conferencing tool contains multiple models that process and transform an input data stream. Transcription, background blurring and noise cancellation models feature within the tool. | ATRS 3.0  Section Tier 2.4.1 |
| Model name: Provide the name of the model. | ATRS 3.0  Section Tier 2.4.2 |
| Model version: Provide the version of the model. | ATRS 3.0  Section Tier 2.4.2 |
| Model task: Provide a short description of the task the model is designed to perform. | ATRS 3.0  Section Tier 2.4.2 |
| Model input: Provide a short specification of the model input. | ATRS 3.0  Section Tier 2.4.2 |
| Model output: Provide a short specification of the model output. | ATRS 3.0  Section Tier 2.4.2 |
| Model architecture: Describe the model architecture. At minimum, please enter: the type of model (e.g. random forest classifier, convolutional neural network, transformer, etc.). This can also include a short description of the method(s) and optimisation(s) employed, or a link to resources providing further resources on the method(s).  Where appropriate, please also describe the model in more detail (e.g. the number of weights and layers, organisation of layers and structural features of note). | ATRS 3.0  Section Tier 2.4.2 |
| Model performance: Provide details on the model's performance. Useful metrics to consider are: accuracy metrics (such as precision, recall, F1 scores), metrics related to privacy, and metrics related to computational efficiency. Describe any performance thresholds your model should exceed. Include links to graphs and tables where appropriate.  If you have evaluated performance with respect to particular subgroups within your data (i.e. fairness analysis) and taken measures to address these, please detail the results and any measures taken to address issues here. | ATRS 3.0  Section Tier 2.4.2 |
| Datasets: List all the datasets that have been used to develop the model. Please indicate which datasets you have written a Dataset card for, if any. | ATRS 3.0  Section Tier 2.4.2 |
| Dataset purposes: Indicate how each dataset was used during the model development process (e.g. training, validation, testing). | ATRS 3.0  Section Tier 2.4.2 |
| Source data name: Provide the name(s) of the dataset(s) used. | ATRS 3.0  Section Tier 2.4.3 |
| Data modality: Indicate the primary modality of the data. | ATRS 3.0  Section Tier 2.4.3 |
| Data description: Provide a short summary of the information captured by the dataset(s). | ATRS 3.0  Section Tier 2.4.3 |
| Data quantities: Describe the amount of data used for model development. This can include the number of samples and attributes within the dataset(s), or the size of the dataset(s) in bytes. If data was split to train, validate and test a model, please provide the relative sizes of each set. | ATRS 3.0  Section Tier 2.4.3 |
| Sensitive attributes: Provide a list of the sensitive attributes contained within the dataset(s), if any. These may include personal data, protected characteristics or proxy variables. | ATRS 3.0  Section Tier 2.4.3 |
| Data completeness and representative-ness: Provide information on the completeness of the data, including missing data and how representative the data is with respect to a target population. | ATRS 3.0  Section Tier 2.4.3 |
| Source data URL: Provide a URL to the openly accessible data if possible. | ATRS 3.0  Section Tier 2.4.3 |
| Data collection: Provide information on the data collection process. This can include details on the original purpose of data collection and the context in which it was initially collected. If the data was initially collected for different purposes and is now being repurposed, there should be consideration of to what extent the data transferable and suitable for the new context. | ATRS 3.0  Section Tier 2.4.3 |
| Data cleaning: Provide information on data cleaning, including a description of any pre-processing or cleaning of the data conducted by either the supplier or public sector customer. | ATRS 3.0  Section Tier 2.4.3 |
| Data sharing agreements: Provide information on data sharing agreements in place. | ATRS 3.0  Section Tier 2.4.3 |
| Data access and storage: Provide details of who has, or will have, access to this data; how long it is stored; under what circumstances; and who is responsible for its storage. Where sensitive data is being stored and accessed, a description of the mechanisms used to protect the security and privacy of the data should be described, including technical (e.g. de- identification techniques, Privacy Enhancing Technologies) and operational (e.g. role-based access controls) methods. | ATRS 3.0  Section Tier 2.4.3 |
| Impact assessment: Provide the names and a short overview of the impact assessments conducted, date of completion, and if possible a summary of the findings. If available, a publicly accessible link should be provided.  If multiple impact assessments were completed, they should all be listed in this field.  Examples of impact assessments are Data Protection Impact Assessments (DPIAs), Equality Impact Assessments, Algorithmic Impact Assessments. This field also provides an opportunity to list other monitoring and evaluation requirements under relevant legislation. | ATRS 3.0  Section Tier 2.5 |
| Risks and mitigations: List and describe the possible risks that have been identified for the algorithmic tool. Where possible, also give an overview of how the risks have been mitigated | ATRS 3.0  Section Tier 2.5 |
| Use existing standards and recording mechanisms such as the ATRS to communicate information about AI solutions to the general public. ATRS is particularly relevant if your tool is deployed, directly or indirectly, in decision-making processes. | Artificial Intelligence Playbook  Section Transparency and explainability |
| Clearly signpost when AI has been used to create content, is interacting with members of the public, or is used in decision-making processes that impact members of the public. | Artificial Intelligence Playbook  Section Transparency and explainability |
| Put in place evaluation and auditing structures, tracking data provenance, design decisions, training scenarios and processes. | Artificial Intelligence Playbook  Section Transparency and explainability |
| Implement transparency and auditing requirements for suppliers. | Artificial Intelligence Playbook  Section Transparency and explainability |
| Use external resources and emerging best practice, such as data cards and model cards for internal transparency. | Artificial Intelligence Playbook  Section Transparency and explainability |
| Make model outputs as explainable as possible, and avoid using less explainable AI methods in areas where explainability is essential. | Artificial Intelligence Playbook  Section Transparency and explainability |
| Consider the use of open source models, which provide more transparency about data sets, code and training processes. | Artificial Intelligence Playbook  Section Transparency and explainability |
| Describe the purpose of personal data processing activities. | Artificial Intelligence Playbook  Section Lawfulness and purpose limitation |
| Document measures and safeguards put in place, and any residual levels of risk posed by the processing | Artificial Intelligence Playbook  Section Lawfulness and purpose limitation |
| Be transparent about the purpose for processing personal data, retention  periods and third parties involved in the processing activity. | Artificial Intelligence Playbook  Section Transparency and individual rights |
| Be transparent about the existence and nature of automated decision making, using the Algorithmic Transparency Recording Standard (ATRS) where required or on a voluntary basis as best practice. | Artificial Intelligence Playbook  Section Transparency and individual rights |
| Provide a clear explanation of the results these systems produce, following guidance such as the ICO’s Explaining decisions made with AI. | Artificial Intelligence Playbook  Section Transparency and individual rights |
| When AI is used, the submitting organisation and authors should clearly declare its use, explain the choice of method and report how it was used, including human input | NICE  Section 8 |
| The use of AI methods, particularly ‘black box’ models, can introduce challenges for transparent reporting of evidence. When their use is justified, submitting organisations should consider how these methods can be accessibly presented, including appropriate referencing (for example, of AI tools used and suitability assessment) and the use of lay language. When available, consider using tools to support the explainability of AI methods and increase transparency of their application | NICE  Section 10 |
| The use of AI can introduce new risks. These risks should be mitigated by adhering to established guidance and checklists (such as Cochrane, PALISADE, TRIPOD+AI and the Algorithmic Transparency Reporting Standard) during the development, application and reporting of AI, and using AI only in the context of following other relevant best practice guidance recommended by NICE | NICE  Section 11 |
| When using AI methods, submitting organisations should report the risks they identified with doing so (for example, regarding concerns about transparency and bias) and steps they took to address those risks | NICE  Section12 |
| AI methods used for real-world data extraction and curation must be reported, in detail, as part of the data suitability assessment outlined in [NICE’s real-world evidence framework](https://www.nice.org.uk/corporate/ecd9/chapter/overview), making use of reporting tools when possible. | NICE  Section 16 |
| peer reviewers must not input content from our confidential funding applications or reviews into, or use, generative AI tools to develop their peer review critiques or applicant responses to critiques | Joint funders statement |
| any outputs from generative AI tools in funding applications should be acknowledged | Joint funders statement |
| applicants and applications are expected to be transparent where they have used generative AI tools in the development of an application | UKRI |
| Assessors, including reviewers and panellists, must not take into account or speculate within their assessment whether generative AI has been used to develop the application | UKRI |
| Researchers must disclose any use of generative AI on their application form except where it has been used for:   - translating applications from other languages to English and/or - improving the standard of English used in applications | Wellcome |
| Expert reviewers and advisory panel members must not input any content from our confidential grant applications or reviews into, or otherwise use, generative AI tools to develop their expert review feedback. Doing this breaches the confidentiality agreement that expert reviewers and advisory panel members agree to. | Wellcome |
| Correctly and explicitly attribute outputs from generative AI tools in funding applications or research by listing the generative AI source, where practicable, naming the specific model/s used and software, and specifying how content was generated (such as listing the prompt used). | Cancer research UK |
| CRUK peer reviewers must not:   - Input any content from a CRUK funding application into generative AI tools. This content is provided to you by CRUK in your capacity as a peer reviewer and sharing with a generative AI tool constitutes a breach of CRUK’s peer review confidentiality and research integrity requirements. - Use generative AI tools in formulating or editing your peer review critiques for funding applications or reviews. CRUK is revising its confidentiality agreements for peer reviewers to clarify this prohibition, which relates to confidentiality and intellectual property concerns in the sharing, viewing and use of data inputted into generative AI tools. | Cancer research UK |
| Where AI technology has been used this should be clearly described. | BMJ Publishing  Section  The Policy |
| We expect authors or others who are creating content to disclose and describe use of AI technologies in (a) any content which is submitted to us, (b) any other work by the authors which underpins or is otherwise connected with the content submitted to us, and (c) any key sources which are cited, to the best of the authors’ knowledge. | BMJ Publishing  Section  Transparent reporting of AI technologies in content submitted to BMJ journals |
| To ensure transparent declaration of AI, authors should Include an acknowledgement of AI use in the ‘contributor’ section of written material. If the AI use was in the course of research, a fuller description should be included in the methods section.  Transparent declaration includes a description of:   - What AI technology was used (the name of the technology) - Why this AI technology was used (the reason for its use) - How the AI technology was used (what the task of the technology was) - Consider including a summary of the input, output, and the way in which the AI output was reviewed on the part of the authors as supplementary files or additional information for the editor to review. The editor may ask for more information and/or for information to be added to the content for internal use and/or for publication. | BMJ Publishing  Section  BMJ Journals |
| If reviewers use AI technology to improve word processing and language, they should declare this when submitting their reports. However, reviewers should preserve the confidentiality of the peer review process by not putting unpublished manuscripts that they are reviewing for BMJ Journals (or information about them) into publicly available AI tools where the security of the confidential information cannot be guaranteed. | BMJ Publishing  Section  Peer review |
| If AI tools have been used to generate main text, then this must be clearly disclosed in the acknowledgments. Authors should not list a generative AI technology as a co-author or author of any submitted manuscript. Generative AI technologies cannot be held accountable for all aspects of a manuscript and consequently do not meet the ICMJE criteria required for authorship. | Frontiers  Section  Artificial intelligence: fair use and disclosure policy |
| If the author of a submitted manuscript has used written or visual content produced by or edited using a generative AI technology, this use must comply to all Frontiers guidelines and policies. Specifically, the author remains responsible for checking the factual accuracy of all content created using generative AI technology. This includes, but is not limited to, any quotes, citations, or references. | Frontiers  Section  Artificial intelligence: fair use and disclosure policy |
| If the author of a submitted manuscript has used written or visual content produced by or edited using a generative AI technology, such use must be acknowledged in the acknowledgments section of the manuscript and the methods section, if applicable. This explanation must list the name, version, model, and source of the generative AI technology. | Frontiers  Section  Artificial intelligence: fair use and disclosure policy |
| We encourage authors to upload all input prompts provided to a generative AI technology and outputs received from a generative AI technology in the supplementary files for the manuscript. | Frontiers  Section  Artificial intelligence: fair use and disclosure policy |
| Generative AI technologies should not be used to review the content of a submitted manuscript or used to make decisions as to the acceptance or rejection of a manuscript. Responsibility for the integrity of the review process must remain with the editors and reviewers who have accepted their roles as relevant experts. | Frontiers  Section  Artificial intelligence: fair use and disclosure policy |
| Authors must confirm that the AI Technology does not claim ownership of their content or impose limitations on its use, as this could interfere with the author’s rights or Wiley's rights, including Wiley’s ability to publish a submission following acceptance. Authors should periodically revisit the AI Technology terms to ensure continued compliance. | Wiley  Section  Artificial intelligence: General Guidance & Best Practice |
| Authors may only use AI Technology as a companion to their writing process, not a replacement. As always, authors must take full responsibility for the accuracy of all content, and verify that all claims, citations, and analyses align with their expertise and research. Before including AI-generated content in a journal submission, authors must carefully review it to ensure the final work reflects their expertise, voice, and originality while adhering to Wiley's ethical and editorial standards. | Wiley  Section  Artificial intelligence: General Guidance & Best Practice |
| Authors should maintain documentation of all AI Technology used, including its purpose, whether it impacted key arguments or conclusions, and how they personally reviewed and verified AI-generated content. Authors must also disclose use of AI Technologies upon submission to a Wiley journal. | Wiley  Section  Artificial intelligence: General Guidance & Best Practice |
| If an author has used AI Technology to develop any portion of a manuscript, its use must be described, transparently and in detail, in the Methods section (or via a disclosure or within the Acknowledgements section, as applicable). | Wiley  Section  Artificial intelligence: General Guidance & Best Practice |
| AI Technology should be used only on a limited basis in connection with peer review. A GenAI tool can be used by an editor or peer reviewer to improve the quality of the written feedback in a peer review report. This use must be transparently declared upon submission of the peer review report to the manuscript’s handling editor | Wiley  Section  Artificial intelligence: General Guidance & Best Practice |
| AI use must be declared and clearly explained in publications such as research papers, just as we expect scholars to do with other software, tools and methodologies. | Cambridge University Press  Section  AI Contributions to Research Content |
| If AI tools played a substantial role in the review process (eg, primary screening, data extraction), consider indicating the use of AI assistance in the title or subtitle. | PRISMA-trAIce  T1 title |
| Briefly summarize the AI tool(s) used, the SLR stage(s) at which they were applied, and their primary role. | PRISMA-trAIce  A1 abstract |
| Briefly state the rationale for choosing to use AI tools for specific tasks in the review (eg, managing large volume of literature, enhancing efficiency, exploring novel methods). | PRISMA-trAIce  I1 introduction |
| If specific AI tools or AI-assisted methods were pre-specified in the review protocol, state this and provide details of where the protocol can be accessed. Report any deviations from the protocol regarding AI use. | PRISMA-trAIce  M1 protocol and registration |
| For each AI tool or system used: a. Specify the name, version number (if applicable), and developer/provider. b. Provide details on how the tool can be accessed (eg, URL, software repository, commercial availability, local instance). c. If a custom-developed AI tool or script was used, describe its core functionality and how it can be accessed or replicated (eg, link to code repository, dataset or base model of a given fine-tuning process). | PRISMA-trAIce  M2 identification and access |
| For each AI tool, clearly describe: a. The specific SLR stage(s) where it was applied (e.g., search, screening, data extraction, Risk of Bias assessment, synthesis, drafting). b. The precise task(s) the AI was intended to perform at each stage. | PRISMA-trAIce  M3 purpose and stage of application |
| Describe the input data provided to each AI tool for its operation: a. For tools that learn or are fine-tuned: Describe the origin, nature, and preparation of any data used for training, fine-tuning, or calibration specific to this review. b. For tools applied to review data: Describe the data fed into the tool (eg, search results, abstracts, full-text articles, specific datasets for analysis). | PRISMA-trAIce  M4 input data |
| Describe the output data generated by each AI tool. This description must include the format of the output (eg, structured JSON, plain text, classification labels with confidence scores) and detail any automated post-processing steps applied to the raw output before it was presented for human review or use. | PRISMA-trAIce  M5 output data |
| For each LLMb/GenAIc tool used, report: a. The full prompt(s) employed for each specific task. If prompts are extensive, provide a detailed description of their structure, key instructions, context provided (eg, inclusion/exclusion criteria, PICOd elements), and any few-shot examples used. Indicate where full prompts can be accessed (eg, supplementary material, repository). b. Key parameters influencing output (eg, model temperature, max tokens, top-p). c. Describe any iterative refinement process for the prompts based on initial outputs or pilot testing. | PRISMA-trAIce  M6 prompt engineering |
| For AI tools other than LLMs/GenAI (or in addition to prompts for them), describe: a. Key algorithms or models employed by the tool (if known and relevant). b. Specific settings, parameters, or configurations used that could influence performance (eg, thresholds for classification in screening tools, active learning model parameters). | PRISMA-trAIce  M7 operational details and setting |
| Describe the process of human interaction with and oversight of the AI tool(s) at each stage: a. How many reviewers interacted with/validated the AI outputs for each task? b. Did reviewers work independently when validating AI outputs? c. What were the qualifications or training of reviewers for AI-assisted tasks? d. How were AI outputs presented to reviewers? e. What proportion of AI outputs were manually reviewed/verified? f. How were discrepancies between AI and human reviewers, or among multiple human reviewers, resolved? g. Describe any processes for calibrating human reviewers or the AI tool. f. Describe the standard procedure for human verification of AI-generated outputs | PRISMA-trAIce  M8 human-AI interaction and oversight |
| Describe methods used to evaluate the AI tool(s) performance for the specific tasks within the review (if applicable and feasible). This may include: a. The reference standard used for evaluation (eg, consensus human decisions). b. The metrics used (eg, accuracy, sensitivity, specificity, precision, recall, F1-score). c. Analyzes conducted to access model bias or rate of erroneous ouputs d. Any pilot testing or validation phases conducted prior to full implementation. | PRISMA-trAIce  M9 AI performance evaluation methods |
| Describe how data handled by AI tools (input, output, intermediate data) was managed and stored, and any measures taken to ensure data privacy, security, and compliance with copyright or terms of service, especially when using third-party cloud-based AI tools. | PRISMA-trAIce  M10 data governance and ethics |
| The PRISMA flow diagram and text clearly distinguish between records/reports excluded or included based on AI tool decisions versus human reviewer decisions at each screening stage where AI was used. Report the number of records processed by AI and the outcomes of that processing. | PRISMA-trAIce  R1 study selection AI assisted |
| Report the results of any performance evaluations of the AI tool(s) for the specific tasks within the review (as described in P-trAIce M9). Include quantitative results (see M9) and measures of agreement between AI and human reviewers if assessed. | PRISMA-trAIce  R2 AI performance metrics results |
| Discuss any limitations encountered in using the AI tool(s) (eg, technical issues, biases identified, challenges in prompt engineering, unexpected outputs, limitations in AI performance for specific sub-tasks). Discuss how these limitations might have influenced the review process or findings. | PRISMA-trAIce  D1 limitations of AI use |
| Briefly discuss the experience of using AI tools in the review, including any perceived benefits (eg, efficiency gains, ability to handle larger datasets) or challenges. Reflect on the usability of the AI tools and the implications for future similar reviews. | PRISMA-trAIce  D2 implications of AI use |
